# Reactive nodular fibrous pseudotumor of the omentum: a rare case report and literature review

**DOI:** 10.1186/s12876-026-04888-1

**Published:** 2026-05-08

**Authors:** Xiujuan Sun, Chengyu Hu, Sisi Wang, Weihua Gong

**Affiliations:** 1 Department of Radiology, The Second Affiliated Hospital of Zhejiang, University School of Medicine, Hangzhou, China; 2 Department of Surgery, The Second Affiliated Hospital of Zhejiang, University School of Medicine, Hangzhou, China; 3 Department of Pathology, The Second Affiliated Hospital of Zhejiang, University School of Medicine, Hangzhou, China

**Keywords:** Reactive nodular fibrous pseudotumor, Omentum, Imaging, Histopathology, Differential diagnosis

## Abstract

**Background:**

Reactive nodular fibrous pseudotumor is a rare benign fibroinflammatory lesion of the gastrointestinal tract and mesentery that can closely mimic malignant disease on clinical examination and imaging. Most reported cases are associated with prior abdominal surgery, trauma, or inflammation. Omental involvement is particularly uncommon, and preoperative diagnosis remains difficult because of its rarity and the limited description of multimodality imaging findings. We report a rare case of omental reactive nodular fibrous pseudotumor in a young man without any history of abdominal surgery, trauma, or abdominal discomfort, highlighting its unusual presentation and diagnostic features.

**Case presentation:**

A 31-year-old man presented with a palpable abdominal mass that had been present for more than 1 month. He had no previous abdominal surgery, trauma, or gastrointestinal symptoms. Contrast-enhanced computed tomography revealed multiple well-defined solid masses in the greater omentum with scattered calcifications. The lesions were similar in attenuation to skeletal muscle on unenhanced images and showed mild progressive enhancement after contrast administration. Magnetic resonance imaging demonstrated low signal intensity on both T1-weighted and T2-weighted images with a similar gradual enhancement pattern. Ultrasound and contrast-enhanced ultrasound showed hypoechoic lesions with limited enhancement. Because malignancy could not be excluded radiologically, surgical resection was performed. Histopathological examination demonstrated spindle cell proliferation within dense hyalinized collagenous stroma with focal chronic inflammatory infiltrates. Immunohistochemical findings supported the diagnosis of reactive nodular fibrous pseudotumor. The patient recovered well after surgery and remained recurrence-free during more than 4 years of follow-up.

**Conclusions:**

This case indicates that reactive nodular fibrous pseudotumor should be included in the differential diagnosis of solid omental masses, even in patients without a history of abdominal surgery or inflammation. Multimodality imaging may provide important clues to its fibrous nature, but definitive diagnosis still depends on histopathological confirmation. Greater awareness of this rare entity may reduce misdiagnosis and help avoid unnecessary aggressive treatment.

## Background

Reactive nodular fibrous pseudotumor (RNFP) is an uncommon, benign lesion of postinflammatory origin characterized by fibroblastic/myofibroblastic proliferation embedded in dense collagenous stroma. First described in 2003, it has since been recognized as a distinct pathological entity. RNFP is often associated with previous abdominal surgery or inflammation and typically requires surgical resection for definitive diagnosis and treatment. Due to its rarity and nonspecific imaging features, it is frequently misdiagnosed preoperatively.

Here, we present a case of RNFP occurring in a young adult male with no identifiable history of abdominal trauma or intervention. This case highlights the imaging and pathological features of RNFP and expands current knowledge by providing a comprehensive multimodal imaging assessment. A review of previously reported cases is included to aid clinicians and radiologists in the recognition and differentiation of this rare entity.

## Case presentation

**Fig. 1:**
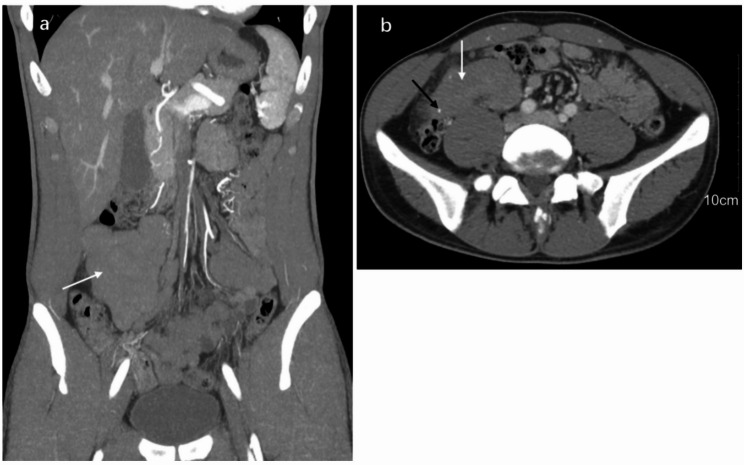
CT image of RNFP. The irregular masses were partially fused (white arrow), with calcifications(black arrow), and exhibited progressively mild enhancement, with minimal enhancement in the arterial phase and gradually increased enhancement in the portal venous and delayed phases (**a**, **b**)

**Fig. 2:**
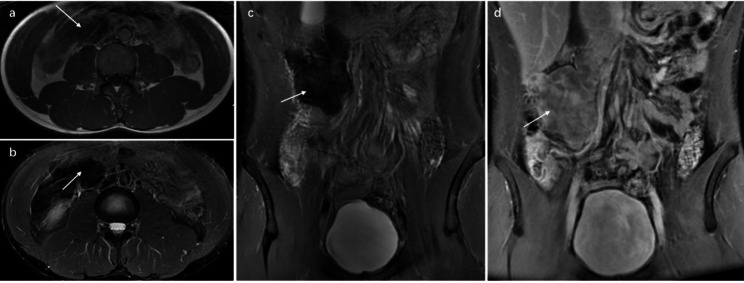
MRI image of RNFP. The irregular masses (white arrow) showed moderate hypointensity on axial T1-weighted MR images (**a**), and significant hypointensity on axial and coronal T2-weighted MR images (**b**, **c**). They exhibited progressively mild enhancement, with minimal enhancement in the arterial phase and gradually increased enhancement in the portal venous and delayed phases on postcontrast MRI (**d**)

**Fig. 3:**
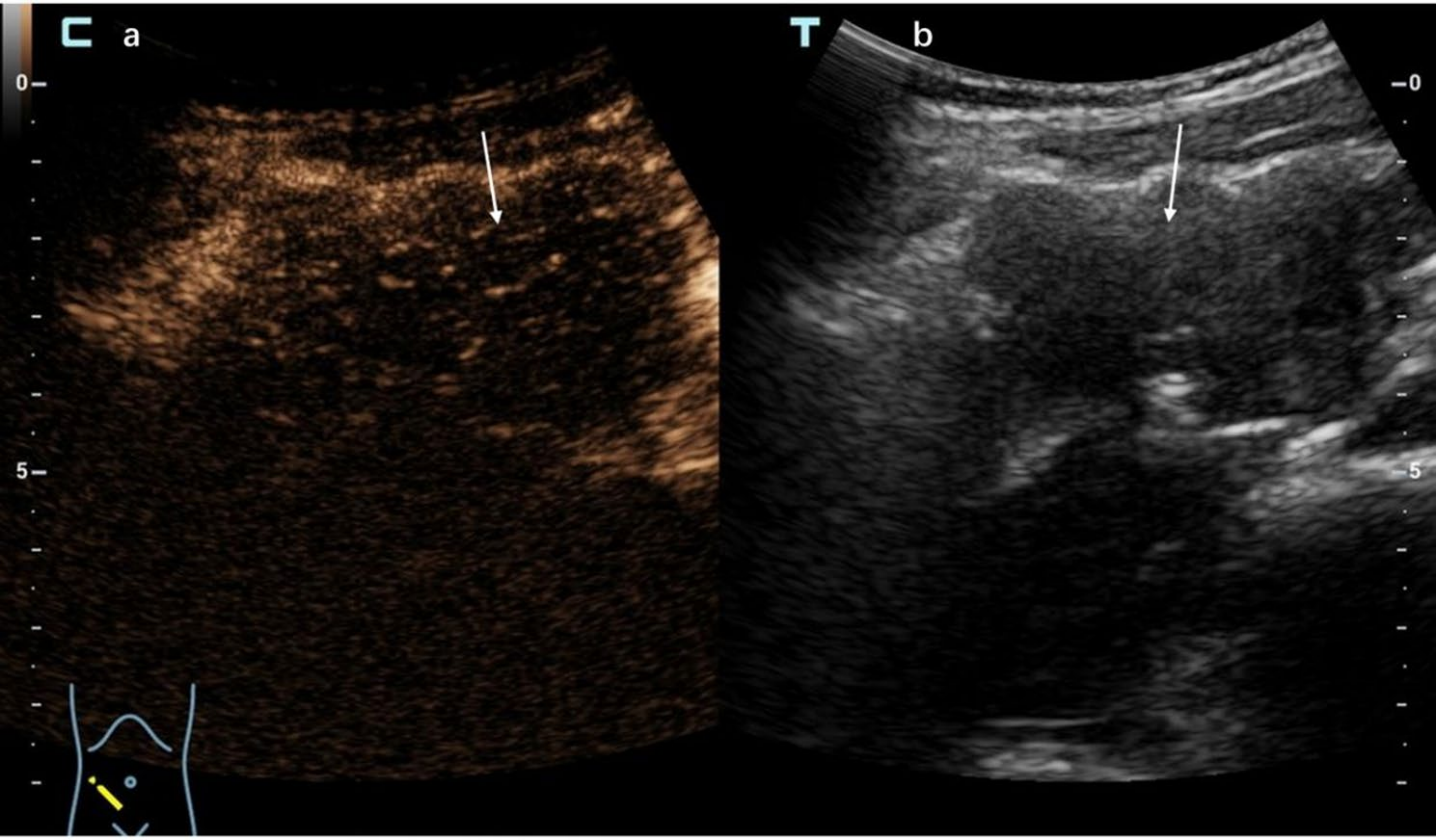
**a** US and **b** CEUS revealed hypoechoic masses (white arrow). On CEUS, no significant arterial-phase hyperenhancement was observed, only mild enhancement was noted in the late phase

**Fig. 4:**
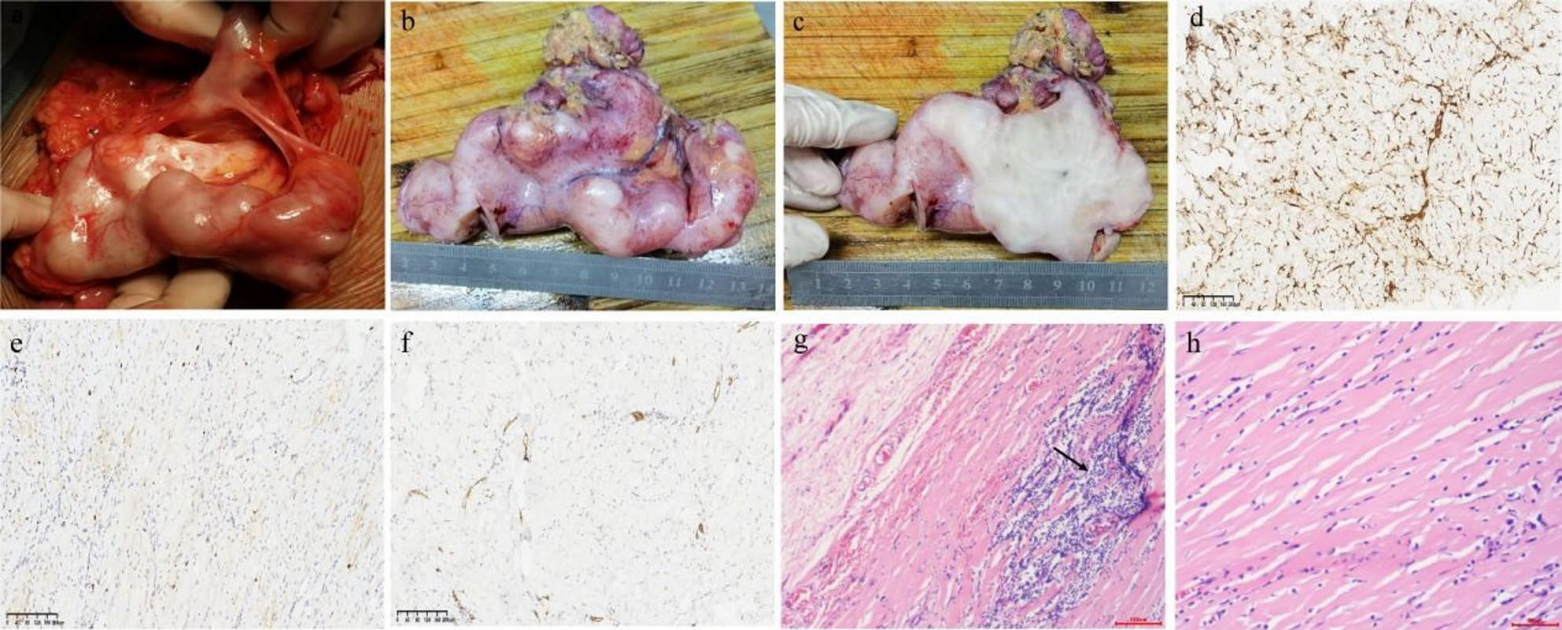
**a**, **b** The lesions were irregular, well-circumscribed, firm, the largest one had a maximum diameter of about 12cm. **c** Serial sections of the mass were tan-white in color. **d** Immunohistochemistry results showed positive vimentin. **e** Immunohistochemistry results showed positive Ki67, proliferation index 5%. **f** Immunohistochemistry results showed negative SMA. (IHC ×100) (**g**, **h**) Histological examination of the H&E revealed that the tissue was composed of proliferative fibrous tissue with marked hyalinization, and focal chronic inflammatory cell infiltrates, predominantly consisting of lymphocytes (black arrow). (H&E, ×100, ×200)

A previously healthy 31-year-old male with no history of abdominal surgery or abdominal pain accidentally palpated an abdominal mass that had been present for over one month. He initially presented to another hospital, where a non-contrast abdominal CT scan revealed multiple soft-tissue masses in the greater omentum. For further diagnostic evaluation and clinical management, the patient was referred to our hospital. On physical examination, a firm, mildly tender mass was palpated on the right side of the abdomen, with no other clinical symptoms. Laboratory test results were within normal limits. To obtain a more comprehensive assessment of the lesions, contrast-enhanced CT and MRI examinations were performed at our institution. CT confirmed the presence of multiple, well-defined, solid omental nodules and masses, the largest measuring approximately 12.1 × 4.6 × 9.7 cm. On non-contrast CT, the irregular masses appeared isodense to skeletal muscle, partially fused, and contained scattered calcifications. Post-contrast images demonstrated mild, progressive enhancement (Fig. 1). MRI revealed moderate hypointensity on T1-weighted images and marked hypointensity on T2-weighted images, with an enhancement pattern similar to CT (Fig. 2). Ultrasound (US) and contrast-enhanced ultrasound (CEUS) showed hypoechoic masses with limited contrast uptake (Fig. 3a–b). This case highlights imaging and immunohistochemical features that may assist in distinguishing RNFP from Gardner fibroma and gastrointestinal stromal tumor (GIST). However, the patient had no personal or family history of Gardner’s syndrome, and gastrointestinal endoscopy revealed no abnormalities. Surgical exploration identified multiple well-circumscribed, firm omental masses, the largest measuring approximately 12 cm (Fig. 4a–b). Gross examination showed tan-white, solid, whorled cut surfaces (Fig. 4c). Histopathological analysis revealed spindle cell proliferation within a collagen-rich, hyalinized stroma, accompanied by scattered lymphocytic infiltrates (Fig. 4g–h). Immunohistochemistry showed positivity for cytokeratin AE1/AE3, vimentin, Bcl-2, and Ki-67 (5% proliferation index), and negativity for SMA, Desmin, CD34, and β-catenin (Fig. 4d–f). These findings confirmed the diagnosis of RNFP. The patient remained recurrence-free at over four years of follow-up. The reporting of this case follows the CARE Statement and associated checklist, which provide evidence based recommendations for high quality case reports.

## Discussion

Since its initial description by Yantiss et al. in 2003, with additional cases documented in subsequent studies, only 28 cases of RNFP have been reported in the scientific literature [1–17]. Our case represents the 29th reported instance of this lesion in the English literature. Among the 28 patients reviewed in the literature (Table 1), 17 were male (60.7%) and 11 were female (39.3%). Of these, 24 cases involved adults (85.7%), with the remaining cases including one in a 1-year-old boy [4] and three in teenagers [10, 12, 16]. The average age of patients was 47.6 years, and our case involved a 31-year-old male, further supporting the observation that adult males are more predisposed to this condition. Clinically, 12 patients (42.9%) presented with abdominal symptoms as the primary manifestation, among whom five experienced acute abdominal pain. Many authors have emphasized the importance of investigating patients’ medical histories, particularly prior abdominal surgeries, as RNFP is believed to arise as an inflammatory response to antecedent abdominal trauma or surgical intervention. Indeed, 11 patients (39.3%) had a documented history of abdominal surgery. Additionally, significant abdominal medical histories were noted in eight cases (28.6%), including duodenal bulbar ulcer (1 case), perforated duodenal diverticulum (1 case), endometriosis (2 cases), chronic bowel obstruction complicated by an external fistula (1 case), ileus caused by a tumor of the ileum (1 case), and ingestion of a foreign body (2 cases). These findings suggest that past abdominal medical or surgical history plays a crucial role in the development of RNFP. However, our case is among the minority of RNFP cases that occur in patients without a prior abdominal operation or other identifiable inciting factors. Moreover, the patient in our case was asymptomatic, with no abdominal discomfort. Thus, similar to a small number of other reported cases, the precise etiology of RNFP in this instance remains unknown.

**Table 1:** Summary of reported cases of RNFP

Author reference/Year	Age(y) /Sex	Clinical history	Location	Multiple / Solitary	CT imaging features	MR imaging features	Tumor great diameter (cm)	Follows-up / Recurrence
Yantiss et al/2003 [1]	48/ M	SH— necrotizing pancreatitis	Mesentery and jejunum	Multiple	N/A	N/A	6.5	Yes/No
	50/F	SH—MEN-1 and multiple abdominal surgeries	Peripancreatic	Solitary	N/A	N/A	4.3	Yes/No
	53/M	AAP	Mesentery and serosa of colon, ileum, vermiform appendix, and right colon	Multiple	N/A	N/A	5.5	Yes/No
	57/M	AAP	Mesentery and serosa of distal ileum	Multiple	N/A	N/A	6.5	Yes/No
	71/M	SH— cholecystectomy and resection of duodenal gastrinoma	Mesentery of transverse colon	Solitary	N/A	N/A	2.8	Yes/No
Zardawi et al/2004 [3]	72/F	SH—cholecystectomy and a strangulated abdominal hernia	Small bowel and the omentum	Multiple	N/A	N/A	2.2	N/A
Chatelain et al/2004 [2]	32/M	Epigastric complaints experienced of abdominal pain, perforated duodenal diverticulum	Ascending colon and duodenum	Solitary	N/A	N/A	9.0	N/A
Daum et al/2004 [4]	59/M	SH—underwent a gastric resection	Small bowel	Solitary	N/A	N/A	3.0	One patient is a recent case and in 3 cases the follow-up is not available. 4 patients with follow-up were without signs of recurrence and metastatic disease 5, 5, 6, and 7 years, respectively.
	46/M	N/A	Sigmoid colon	Solitary	N/A	N/A		
	1/M	AAP	Appendix	Solitary	N/A	N/A	3.0	
	68/M	N/A	Cecum	Solitary	N/A	N/A	10.0	
	30/F	Chronic bowel obstruction complicated by external fistula	Small intestine, terminal ileum, cecum and peritoneum	Multiple	N/A	N/A	8.0	
	65/F	N/A	Subserosa and mesocolon of large intestine	Solitary	N/A	N/A	8.0	
	22/M	N/A	Ileum and omentum	Solitary	N/A	N/A	7.0	
	41/M	N/A	Sigmoid colon	Solitary	N/A	N/A	6.0	
Saglam et al/2005 [5]	28/F	AAP	Bilateral ovarian surfaces, appendix, bowel mesentery, abdominal peritoneal wall and the omentum	Multiple	N/A	N/A	6.0	N/A
Gauchotte et al/2009 [6]	60/M	Duodenal bulbar ulcer associated with antral gastritis and autoimmune polyendocrinopathy	Three lesions in the gastric wall and an adjacent lesion in the lesser omentum	Multiple	Isodense on unenhanced CT, with progressive contrast enhancement, no calcifications	Moderate hypointensity on T1 and significant hypointensity on T2-weighted images	2.2	Yes, 4 months/No
Yin et al/2011 [7]	65/M	AAP	Mesentery	Solitary	A heterogeneous mesenteric mass with calcications	N/A	3.0	N/A
Virgilio et al/2012 [8]	71/M	SH— colon adenocarcinoma	Ileal loop, hepatic surface, left paracolic gutter	Multiple	N/A	N/A	6.0	Yes, 4 years/No
Tam, et al/2012 [9]	54/F	SH—laparoscopic appendicectomy and cholecystectomy, right upper quadrant abdominal pain	Hepatic hilum	Solitary	N/A	N/A	4.0	N/A
McAteer et al/2012 [10]	13/F	AAP	Attached at one end to the antimesenteric border of the jejunum via vascular pedicle and adherent to the pelvic wall at its distal end	Solitary	Heterogeneous mass	N/A	8.8	N/A
Salihi et al/2014 [11]	45/F	Intractable menometrorrhagia, pain, and gradual abdominal swelling	Omentum, pelvic wall, right ovary, sigmoid, and Douglas pouch	Multiple	Heterogeneous iso- to hypoattenuating masses with punctiform calcifications	Polylobular masses with very low signal intensity on T1WI and T2WI, strongly hypovascular with peripheral rim-like enhancement	7.0	Yes/No
Yi et al/2014 [12]	16/F	progressive epigastric discomfort of 3 months	Stomach	Solitary	Isodensity on unenhanced CT and homogenous moderate enhancement	N/A	8.0	Yes, more than 2 years/No
Yan et al/2015 [13]	60/F	SH— leiomyoma of the uterus, AAP	Mesentery, greater omentum and serosal surface of the colon	Multiple	N/A	N/A	10.0	Yes, 12 months/No
Ciftci et al/2015 [14]	71/M	SH— colon adenocarcinoma	Between the diaphragm, transverse colon and stomach	Solitary	N/A	Homogeneous, low signal intensity mass	19.5	Yes, 8 months/No
Moodley et al/2018 [15]	65/F	SH— GIST	In the gastrohepatic ligament, attached to the adventitia of the stomach	Solitary	N/A	N/A	1.8	N/A
Girsowicz et al/2018 [16]	17/M	AAP	Transverse mesocolon and peritoneum	Multiple	Inhomogeneous calcified mass associated with rosary bead enhancement of its wall after contrast injection	N/A	10.0	Yes, 5 years/No
Chen et al/2022 [17]	54/M	SH—radical distal gastrectomy for gastric cancer	Closely adhered to the spleen artery and back wall of the gastrointestinal anastomosis	Solitary	The lesion with well-defined isodensity and no necrosis, slight enhancement	N/A	5.5	Yes, 28 months/No

*M* Male, *F* Female, *SH* Surgery history, *N/A* Not available, *AAP* Acute abdominal pain

RNFP typically originates from the subserosal surface of the bowel or within the adjacent mesentery and is frequently associated with local injury or inflammation [14]. The most commonly involved anatomic sites include the mesentery (10 cases), small bowel (8 cases), colon (7 cases), omentum (7 cases), appendix (3 cases), peritoneum (3 cases), gastric wall (2 cases), cecum (2 cases), ovary (2 cases), and duodenum, hepatic surface, hepatic hilum, gastrohepatic ligament, and peripancreas (1 case each). RNFP most often presents as a solitary mass (17 cases, 60.7%), although a minority of cases (11 cases, 39.3%) exhibit multiple masses. Notably, our patient presented with multiple abdominal masses localized exclusively to the omentum. We speculate that the pathogenesis in this case may be related to the patient having a history of occult microtrauma or other subclinical abdominal inflammatory processes.

Microscopically, RNFP is characterized by cellular proliferation of spindle cells within a hyalinized, dense collagenous background. Inflammatory cell infiltration, predominantly lymphocytes frequently arranged in lymphoid aggregates, is a consistent feature reported in most cases. Immunohistochemical analysis typically reveals positive staining for cytokeratins (AE1/AE3), vimentin, and smooth muscle actin (SMA) [4, 6]. In our case, spindle cells were positive for cytokeratins (AE1/AE3), vimentin, Bcl-2, and Ki67 but were negative for SMA. Immunohistochemical analysis showed negative staining for SMA, which is contrary to the typical SMA positivity described in the literature for RNFP. It is important to note that immunophenotypic variability exists in RNFP, and the expression of certain markers, including SMA, can vary between cases. Therefore, the diagnosis of RNFP should be based on a comprehensive assessment of histological, immunohistochemical, and clinical findings, rather than relying solely on a single marker. Although SMA positivity is commonly reported in the literature, the expression of SMA may not be universally present, and this variability should be considered when diagnosing RNFP.

Among the 28 patients reviewed in the literature, only 7 patients (25%) underwent CT scans, and 3 patients (10.7%) underwent MRI scans. Radiological descriptions of RNFP are limited. The imaging features are as follows: Lesions appear isodense on non-contrast CT scans, sometimes with calcifications. On MRI, they show homogeneous hypointensity on T1-weighted images and marked hypointensity on T2-weighted images, with progressive mild enhancement. US and CEUS revealed hypoechoic masses with mild contrast media uptake. To our knowledge, this case represents one of the most comprehensively imaged RNFPs reported to date. In our patient, the masses appeared isodense on non-contrast CT with calcifications, demonstrating progressive mild enhancement on postcontrast CT. MRI revealed moderate hypointensity on T1-weighted images and marked hypointensity on T2-weighted images. These imaging characteristics strongly suggest fibrotic tissue. The enhancement pattern observed on MRI was consistent with that seen on CT. The imaging findings in our patient align well with those previously described in the literature.

Cases of RNFP originating in the omentum are exceptionally rare. Accurate differentiation of RNFP from other abdominal reactive processes is crucial, including nodular fasciitis, calcifying fibrous pseudotumor, sclerosing mesenteritis, intra-abdominal fibromatosis, and more aggressive neoplasms such as GIST, intra-abdominal inflammatory myofibroblastic tumors, and inflammatory fibrosarcoma [12]. In our case, the initial differential diagnosis included GIST and Gardner fibroma. Gardner Fibroma is a benign fibrous tumor that typically arises in the skin or soft tissues. It is most commonly associated with Gardner’s syndrome, a genetic condition that predisposes individuals to multiple types of tumors. Both Gardner fibroma and desmoid-type fibromatosis arise from fibrous tissue, and they share some similar clinical and histological features. However, desmoid tumors are more aggressive, with potential for infiltration into surrounding structures, whereas Gardner fibromas are usually benign and confined to the soft tissues without infiltrative growth. GIST, the most common mesenchymal tumors of the gastrointestinal tract, typically demonstrate distinct CT characteristics: well-defined margins, large hypervascular enhancing masses with heterogeneous enhancement patterns due to necrotic and hemorrhagic components, and potential local invasion [18]. On MRI, the solid components of GIST typically appear hypointense on T1-weighted imaging, hypo- to iso-intense on T2-weighted imaging, and demonstrate moderate to marked contrast enhancement [19]. Histologically, GIST exhibit greater cellularity compared to RNFP and are immunohistochemically positive for DOG1, CD117, and CD34 [13, 20]. Gardner fibroma, characterized by hypocellular, densely collagenous tissue resembling normal fibrous tissue or scar, consistently expresses CD34 [21]. However, our patient showed no clinical evidence of Gardner’s syndrome, with negative personal and family history, and unremarkable gastrointestinal endoscopy findings.

Surgical resection remains the primary and effective treatment modality for RNFP. Among the 28 reviewed cases, complete resection was achieved in 25 patients (89.3%), while incomplete resection was performed in 3 cases (10.7%) due to extensive disease with multiple masses or nodules. Follow-up data were available for 17 patients (60.7%), with a follow-up duration ranging from 4 months to 7 years. Our case has maintained disease-free status during the 4-year follow-up period. The prognosis of RNFP is consistently favorable, with no reported cases of recurrence or distant metastasis following surgical intervention.

Nevertheless, the single-case design inherently limits the generalizability of our findings. Future large-scale cohort studies with extended follow-up are required to further validate the diagnostic value and clinical relevance of RNFP.

## Conclusion

RNFP is a rare but benign fibrous lesion that should be considered in the differential diagnosis of omental or mesenteric masses, particularly in patients with a history of abdominal surgery or inflammation. The imaging features are suggestive of RNFP but are not specific, with overlap existing with other fibromas or desmoid-type lesions. Comprehensive imaging, including CT, MRI and CEUS, can aid in preoperative assessment, although definitive diagnosis relies on histopathological evaluation. Awareness of this entity is crucial to avoid misdiagnosis and unnecessary aggressive treatment. Surgical resection remains curative, with an excellent prognosis. 

## Data Availability

All relevant data has been presented in the manuscript and further inquiry can be directed to the corresponding author.

## Abbreviations

RNFP: Reactive nodular fibrous pseudotumor
US: Ultrasound
CEUS: Contrast-enhanced ultrasound
CT: Computed Tomography
GIST: Gastrointestinal stromal tumor
SMA: Smooth muscle actin
